# Lateral Curvature and the Clinic

**Published:** 1912-07-06

**Authors:** 


### THE IDEAL SCHOOL CLINIC.

### Lateral Curvature and the Clinic.

There is often a great deal of difficulty experienced in estimating the degree of lateral curvature present in a school-child. We still lack a useful and convenient method of comparison in slight cases. While every degree of scoliosis does not demand treatment at the clinic, every variety, no matter how slight it may be, calls for advice and home or class treatment. The school doctor should take not only the parents and the teachers into his confidence but the child as well. It may be said that a small child in a lower department cannot possibly be made to understand that a certain attitude is detrimental to his health, but that is quite a mistake.

The writer has found it easy to get even small children to see that curved back was not a "nice" condition. The use of small plaster models—such as the doctor can himself readily make out of the school modelling clay—will help to illustrate the points on which stress should be laid and fix the attention of the children. It is needless to say that the doctor must carefully avoid technicalities. Even the use of the term lateral curvature is liable to give rise to an entirely mistaken impression on the part of parents and teachers who imagine that the children who are diagnosed as "curved backs" are suffering from disease of the spine. It must be carefully explained that here we have not to do with a disease, but with a weakness which may and must be cured. By impressing the child with the ugliness of an absurd carriage, and showing him that such carriage is entirely his own fault, much may be won to further treatment. Those who maintain that the children cannot grasp such advice or follow the reasoning are generally quite unable to explain even the simplest matters to children. They lack the gift of winning confidence and securing the child's attention. Such men and women may make good consultants, but they are useless, and even harmful, in a school and should not become school doctors.

### Its Early Treatment.

The first essential then in treatment is the correction of faults of posture and carriage. These are much more easily corrected at home or in the class-room than in the clinic. In every case the utmost care must be taken, even in cases which seem to be very slight in degree, that the deformity is not due to any real pathological lesion which needs more active treatment or treatment of quite a different kind. Often great harm is done by overlooking such defects as commencing hip disease, coxa vara, flat-foot, or even incipient spinal disease itself or a nervous lesion. The examination of children who are found to suffer from scoliosis needing treatment must therefore be thorough, and it is important to urge that the child must be stripped. When the necessity for such exposure is explained to the parents there is usually no trouble in securing their permission. Every school doctor knows, or ought to know, that such exposure without the parents' consent is an indecent assault on the child, and, however probable it is that such a charge will not be maintained against the examiner in any court of law, its incidence must be prevented by taking that common-sense precaution of asking the parents' permission. When the parents are not present the assumption is that they consent to any examination, though this is a fine legal point which has not been settled yet. With these legal difficulties, however, we shall deal on another occasion.

### Alterations of Furniture.

Having eliminated all possible pathological lesions it remains for the school doctor to prescribe treatment for the scoliosis. The child's seat in the class should be examined and the leg measurements compared with the height of the school bench and the elevation of the desk. By simple alterations easily made in the furniture an abnormal carriage of the body can often be corrected without much trouble. In the case of boys slight degrees of scoliosis are best treated by inducing the child to join a gymnastic class or to carry out easy bending exercises at home. The good results of joining a scout patrol must be admitted by everyone who has seen how excellently the carriage and bearing of boys are influenced by the discipline and exercises of the patrol under a good scoutmaster. With girls the home treatment is apt to be a matter of greater difficulty.

One of the causes of scoliosis in weak girls is "minding the baby," and this is a very difficult and insidious one to counteract. Swinging exercises and the caution to carry the child in different positions are helpful. An easy and good exercise is to carry by balancing a book or light article on the head; the balancing attitude induces an upright carriage. Walking exercises in which the legs are properly lifted are also serviceable, but are usually best done in class. Individual attention is best in every case where the supervisor is not a trained expert. Company or platoon drill should therefore be discouraged. Some consideration of the games in which the child indulges is necessary. Games which mean freedom of action and exercise of the whole body are of the utmost importance. The best of these are water sports, such as water polo, which the swimming classes indulge in. The doctor should do his utmost to induce every boy and girl in his school to learn swimming. At present we believe most School Boards prohibit the masters taking boys under eight years of age to the swimming baths. This is a too cautious policy which should be altered. Games that induce cramped attitudes—one of these is marbles, another is school cricket—should be given up in favour of the more active exercising games.

(*To be continued.*)

